# Integration of Multiple Genomic and Phenotype Data to Infer Novel miRNA-Disease Associations

**DOI:** 10.1371/journal.pone.0148521

**Published:** 2016-02-05

**Authors:** Hongbo Shi, Guangde Zhang, Meng Zhou, Liang Cheng, Haixiu Yang, Jing Wang, Jie Sun, Zhenzhen Wang

**Affiliations:** 1 College of Bioinformatics Science and Technology, Harbin Medical University, Harbin, Heilongjiang, 150081, PR China; 2 Department of Cardiology, The Fourth Affiliated Hospital of Harbin Medical University, Harbin, Heilongjiang, 150001, PR China; Oxford Brookes University, UNITED KINGDOM

## Abstract

MicroRNAs (miRNAs) play an important role in the development and progression of human diseases. The identification of disease-associated miRNAs will be helpful for understanding the molecular mechanisms of diseases at the post-transcriptional level. Based on different types of genomic data sources, computational methods for miRNA-disease association prediction have been proposed. However, individual source of genomic data tends to be incomplete and noisy; therefore, the integration of various types of genomic data for inferring reliable miRNA-disease associations is urgently needed. In this study, we present a computational framework, CHNmiRD, for identifying miRNA-disease associations by integrating multiple genomic and phenotype data, including protein-protein interaction data, gene ontology data, experimentally verified miRNA-target relationships, disease phenotype information and known miRNA-disease connections. The performance of CHNmiRD was evaluated by experimentally verified miRNA-disease associations, which achieved an area under the ROC curve (AUC) of 0.834 for 5-fold cross-validation. In particular, CHNmiRD displayed excellent performance for diseases without any known related miRNAs. The results of case studies for three human diseases (glioblastoma, myocardial infarction and type 1 diabetes) showed that all of the top 10 ranked miRNAs having no known associations with these three diseases in existing miRNA-disease databases were directly or indirectly confirmed by our latest literature mining. All these results demonstrated the reliability and efficiency of CHNmiRD, and it is anticipated that CHNmiRD will serve as a powerful bioinformatics method for mining novel disease-related miRNAs and providing a new perspective into molecular mechanisms underlying human diseases at the post-transcriptional level. CHNmiRD is freely available at http://www.bio-bigdata.com/CHNmiRD.

## Introduction

MicroRNAs (miRNAs) are endogenous small non-coding RNAs (~22nt) that function by binding to the 3’ untranslated regions (3’UTRs) of target mRNAs, and then inhibiting their expression [1, 2]. According to miRBase (Release 21) [3], more than 1800 human miRNAs have been discovered in the last few years. MiRNAs are known to participate in many important biological processes including cell proliferation, differentiation and apoptosis[4]. The dysregulation of miRNA expression is therefore associated with a broad range of diseases [5], such as cardiovascular diseases [6, 7], neurodevelopmental diseases [8–10] and cancers [5, 11, 12]. Identification of disease-related miRNAs will provide novel insights into the molecular mechanisms underlying human diseases at the post-transcriptional level.

Many miRNAs were found to be associated with certain diseases using various biological experiment methods. To provide a mechanism to comprehensively search for these experimentally verified miRNA-disease associations, researchers have constructed several publicly-available and manually-curated databases, such as HMDD [13] and miR2Disease [14]. However, the collection and inclusion of verified miRNA-disease associations in these databases is far from complete, and identifying disease-related miRNAs from the multitude of candidate miRNAs by biological experimentation is time consuming and labor-extensive. Therefore, the development of effective computational methods for inferring miRNA-disease associations at the systematic level is urgently needed.

Computational methods can produce statistically significant results from a large amount of biological data and serve as a powerful tool for guiding further biological experiments. Based on miRNA functional similarity network (MFSN), different algorithms (Jiang’s method [15], RWRMDA [16], NetCBI [17], HDMP [18], RLSMDA [19]) have been developed to predict disease-related miRNAs (S1 Table). For example, Jiang et al. [15] constructed a MFSN by establishing a relationship between two miRNAs based on their significantly shared common targets, and they then integrated the MFSN with a disease network to infer potential miRNA-disease associations. The MFSN they constructed considered the number of overlapping miRNA targets while neglecting the functional link between them, and only the direct neighbor information of each miRNA was utilized in their scoring system. Additionally, this method was not work for disease whose all neighbor diseases are not associated with any known miRNAs. In RWRMDA [16], NetCBI [17], HDMP [18] and RLSMDA [19], the MFSN they adopted was constructed based solely on the information of known miRNA-disease associations using Wang et al.’s method [20]. Moreover, in these methods, the same miRNA-disease relations were used to construct the MFSN and evaluate the performance, which might over-estimate the performance. In addition, RWRMDA and HDMP were not applicable to disease which did not have any known related miRNAs. Recently, based on protein-protein interaction (PPI) networks, Shi et al. [21] developed a computational framework to identify miRNA-disease associations by focusing on the functional link between miRNA targets and disease genes. Additionally, Mork et al. [22] presented a method in which miRNA-disease associations were inferred by integrating miRNA-protein associations and protein-disease associations. However, these two methods neglected to use information of known miRNA-disease associations, which could improve their predictive performance. In contrast, Xu et al. [23] and Jiang et al. [24] constructed different feature vectors and trained a support vector machine classifier for distinguishing positive miRNA-disease associations from negative ones, respectively. But, there were no verified negative microRNA-disease associations, which result in the difficulty or impossibility for collection of negative disease-related miRNAs. Hence, the low-quality negative samples used in these two studies might largely reduce the predictive accuracy. High-throughput technologies have produced huge amounts of genomic data, which can be used in many ways to predict miRNA-disease associations. However, individual sources of genomic data tend to be noisy and incomplete, which downgrades the prioritization algorithms. Therefore, the question of how to effectively integrate different types of genomic data to improve predictive performance is a major challenge.

In this study, we constructed a complex heterogeneous network (CHN) by integrated PPI data, gene ontology (GO) data, miRNA-target relationships, disease phenotype data and known miRNA-disease associations. Based on the CHN, a computational model, CHNmiRD, was developed to identify miRNA-disease associations by performing random walk analysis. The results of cross validation and case studies suggested that CHNmiRD was effective for uncovering unknown miRNA-disease associations.

## Materials and Methods

### Human miRNA-disease associations and miRNA targets

Human miRNA-disease associations were retrieved from HMDD (version 2.0) [13]. This version of HMDD, released in 2013, has recorded 10,368 high-quality, experimentally verified miRNA-disease associations from 3,511 papers. Repeating miRNA-disease entries were removed, miRNA precursors were mapped to mature miRNAs using miRBase, and disease names were curated based on Online Mendelian Inheritance in Man (OMIM) [25] disease ID. Finally, 3,536 miRNA-disease associations involving 370 miRNAs and 105 diseases were obtained (S1 File). These miRNA-disease associations were used to construct a disease phenotype-miRNA network and used as the gold standard dataset for evaluating performance.

The miRNA targets were chosen from three widely used and experimentally validated miRNA target databases: TarBase (version 6.0) [26], miRTarBase (version 4.5) [27] and miRecords (version 4) [28]. We merged these three databases, and after removing miRNAs that have only one target and unifying the name of mature miRNAs based on miRBase, 37,659 targeting pairs involving 402 miRNAs and 12,360 target genes were obtained (S2 File).

### Disease phenotype network and disease phenotype-miRNA network

Disease phenotype similarity scores were calculated by MimMiner [29] which computed a disease phenotype similarity score for two disease phenotypes based on the text mining analysis of their disease phenotype descriptions contained in the OMIM database. For each disease phenotype, the similarity between it and any other disease phenotypes in the OMIM database was computed using MimMiner, and the K most similar disease phenotypes, called K-nearest neighbors (KNN), were identified. The disease phenotype was connected with its KNNs and weighted using the similarity measure calculated by MimMiner. The network constructed by this method was called the KNN graph. In this study, we constructed a disease phenotype network (DPN) using 5-NN network (S3 File) followed some previous studies [30, 31].

As described above, the disease phenotype-miRNA relationships were extracted from the HMDD database (version 2.0) [32]. These relationships can be viewed as a bipartite disease phenotype-miRNA network (DPMN) in which one node is the miRNA, and the other is the disease phenotype, and the edges are the disease phenotype-miRNA relationships. This network can be used as a bridge to construct a CHN (described later).

### MiRNA functional similarity network based on PPI and GO

MiRNA performs its regulatory function primarily through its target mRNA(s), and miRNAs with similar functions tend to target functionally related genes [33]. Therefore, for a given pair of miRNAs, their functional similarity score could be obtained by calculating the functional similarity of their target mRNA set. Firstly, the functional similarity score of two miRNA target sets was calculated based on PPI considering the functional communication and physical interaction between gene sets by using GsNetCom [34]. Secondly, we adopted GSFS [35] to compute the functional similarity score of two miRNA target sets based on three sub-ontologies (biological process, BP; molecular function, MF; and cellular component, CC) of GO. Finally, four miRNA functional similarity matrices were obtained by using different data sources. In order to make use of the global network similarity information, four weighted MFSNs were constructed according to the above miRNA functional similarity matrices, in which the edges were assigned different functional similarity scores between miRNAs.

### Random walk with restart algorithm

Random walk with restart (RWR) is a global network ranking algorithm [36]. The random walker starts from a seed node (or a set of seed nodes, simultaneously) and proceeds to randomly selected neighbors based on the probabilities of the edges between the two nodes. Formally, RWR is an iterative algorithm and defined as follows:

$${\text{P}}_{t+1}=(1-\alpha )M^{\text{T}}{\text{P}}_{t}+\alpha {\text{P}}_{0}$$ (1)

where P_0_ is the initial probability vector, constructed such that equal probabilities are assigned to all of the seed nodes, with the sum of the probabilities equal to 1. P_*t*_ is a vector in which the *i*-th element holds the probability of finding the random walker at node *i* at step *t*. *M* is the transition matrix of the network, in which (*i*, *j*)-th element of *M* denotes the transition probability from node *i* to node *j*, and it is computed as the row-normalized adjacency matrix of the network. *α* is the restart probability of the walker returning to the seed node, the closer the value of *α* is to 0, the more global the view observed.

We performed the algorithm until the probability of all of the nodes reached a steady state, measured by the change between P_*t*_ and P_t+1_ (measured by the L_1_ norm) falling below 10^−10^. The stable probability is defined as P_∞_, which gives a measure of similarity between non-seed nodes and seed nodes.

### Ranking algorithm based on random walk with restart on complex heterogeneous networks

**Fig 1:**
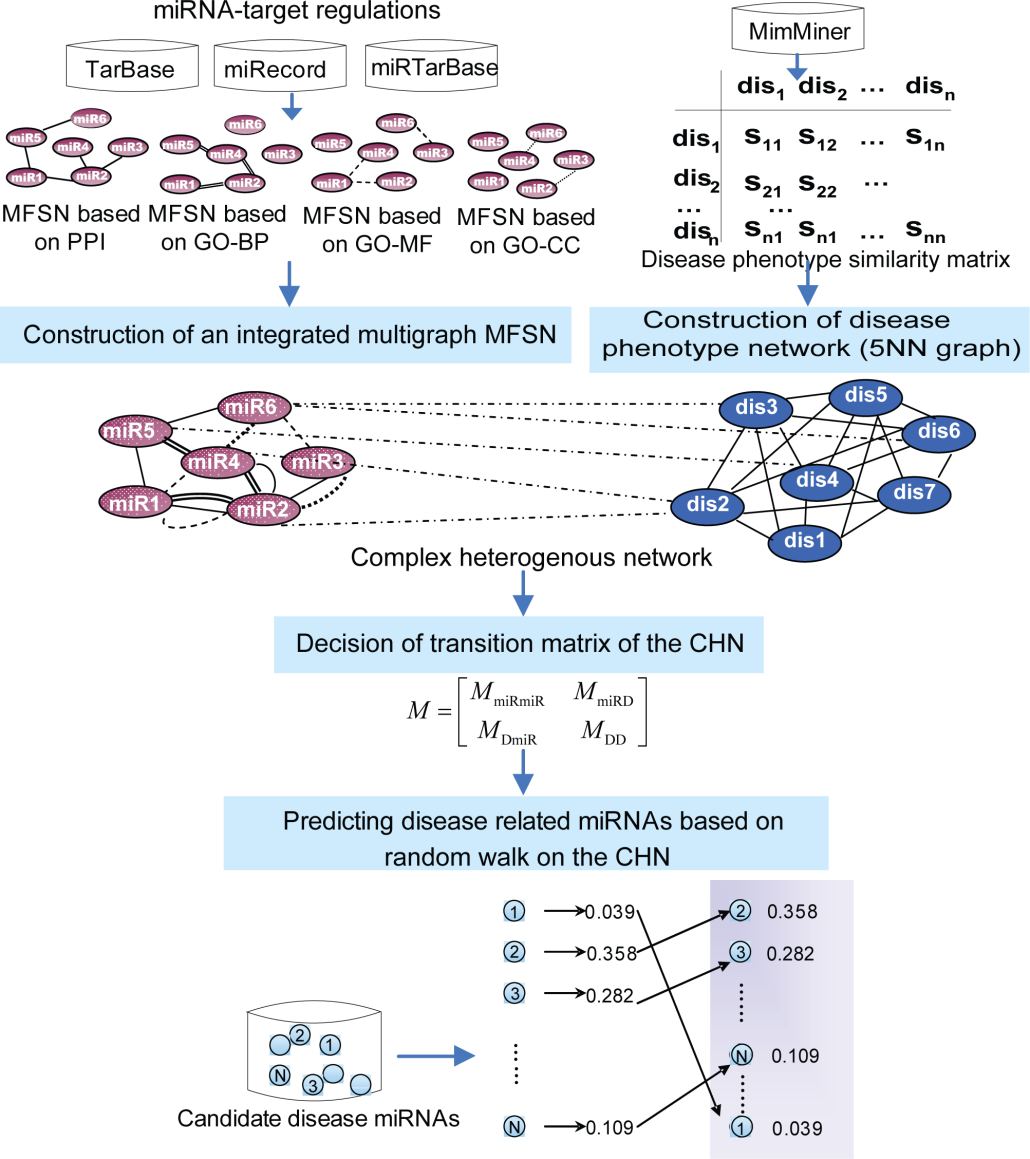
An overview of the CHNmiRD method. Firstly, four MFSNs were constructed based on different genomic data by means of miRNA-target relationships and a disease phenotype network was constructed using the information of disease phenotype similarity. Then the complex heterogeneous network was generated by connecting the disease phenotype network and the integrated multigraph MFSN using the known miRNA-disease relationship information. Finally, the predicting miRNA-disease associations were obtained by implementing RWR algorithm on the complex heterogeneous network.

In this study, we presented a complex heterogeneous network computational model, CHNmiRD, to infer potential miRNA-disease associations by combining an integrated multigraph MFSN and DPN. Our method was an expansion of a previous method for predicting disease-related protein-coding genes [31]. The strategy to identify miRNA-disease associations using CHNmiRD is shown in Fig 1. The main flow of CHNmiRD consists of four steps: (1) constructing an integrated multigraph MFSN; (2) generating the CHN; (3) deciding the transition matrix of the CHN and (4) deciding the initial probability vector of the RWR algorithm to rank candidate disease miRNAs.

As mentioned above, four MFSNs were obtained based on different types of genomic data and were merged into a single multigraph MFSN. On the merged multigraph MFSN, the transition probability from node *i* to node *j* was computed as the expected value of the transition probabilities corresponding to four types of links between node *i* to node *j*. Suppose A^*k*^ is the transition matrix of the network *k* (*k* = 1, 2, 3, 4), and the corresponding (*i*, *j*)-th element of the matrix is A^*k*^(*i*, *j*) denoting the transition probability from node miRNA *i* to node miRNA *j*. The transition probability from node *i* to node *j* on the integrated multigraph MFSN can then be computed as

$$\text{A}(i,j)=\sum_{k=1}^{{\text{N}}_{i}}\omega^{k}{\text{A}}^{k}(i,j)$$ (2)

Where N_*i*_ is the number of networks to which node miRNA *i* is associated. *ω*^*k*^ is the probability of choosing the *k*-th network. Here, we set $\omega^{k}=\frac{1}{{\text{N}}_{i}}$ denoting the selection of any network with equal probability. Thus, an integrated multigraph MFSN could be obtained (S4 File).

A CHN was constructed by connecting a DPN and an integrated multigraph MFSN through the use of the human miRNA-disease associations from the HMDD database. Suppose A_(m×m)_, B_(n×n)_ and C_(m×*n*)_ denote adjacency matrices for the integrated multigraph MFSN, DPN and DPMN, respectively. The adjacency matrix of the CHN can then be represented as $\left[\begin{array}{cc} \text{A} & \text{C}\\ {\text{C}}^{\text{T}} & \text{B} \end{array}\right]$, where C^T^ is the transpose of C.

Next, we computed the transition matrix of the CHN. Suppose the transition matrix of the CHN is $M=\left[\begin{array}{cc} M_{\text{miRmiR}} & M_{\text{miRD}}\\ M_{\text{DmiR}} & M_{\text{DD}} \end{array}\right]$, where *M*_miRmiR_ and *M*_DD_ are transition matrices indicating the probability from one miRNA (disease) to another miRNA (disease) in the random walk, respectively; *M*_miRD_ is the transition matrix from the integrated multigraph MFSN to the DPN, and *M*_DmiR_ is the transition matrix from the DPN to the integrated multigraph MFSN. Let *λ* be the jumping probability, that is, the probability of jumping from the integrated multigraph MFSN to the DPN or *vice versa*. Let *miR*_*i*_ denote the *i*-th miRNA in the integrated multigraph MFSN and *d*_*i*_ represents the *i*-th disease phenotype in the DPN. The transition matrix can thus be defined as follows:

The transition probability from *miR*_*i*_ to *miR*_*j*_ is defined as

$$M_{\text{miRmiR}}(i,j)=p(miR_{j}|miR_{i})=\{\begin{array}{c} \text{A}(i,j)/\sum_{j}\text{A}(i,j)\;\text{if}\;\sum_{j}\text{C}(i,j)=0\\ \left(1-\lambda )\text{A}(i,j\right)/\sum_{j}\text{A}(i,j)\;\text{otherwise} \end{array}$$ (3)

The transition probability from *d*_*i*_ to *d*_*j*_ is defined as

$$M_{\text{DD}}(i,j)=p(d_{j}|d_{i})=\{\begin{array}{c} \text{B}(i,j)/\sum_{j}\text{B}(i,j)\;\text{if}\;\sum_{j}\text{C}(j,i)=0\\ \left(1-\lambda )\text{B}(i,j\right)/\sum_{j}\text{B}(i,j)\;\text{otherwise} \end{array}$$ (4)

The transition probability from *miR*_*i*_ to *d*_*j*_ is defined as

$$M_{\text{miRD}}(i,j)=p(d_{j}|miR_{i})=\{\begin{array}{c} \lambda C(i,j)/\sum_{j}\text{C}(i,j)\;\text{if}\;\sum_{j}\text{C}(i,j)\neq 0\\ \;0\;\text{otherwise} \end{array}$$ (5)

The transition probability from *d*_*i*_ to *miR*_*j*_ is defined as

$$M_{\text{DmiR}}(i,j)=p(miR_{j}|d_{i})=\{\begin{array}{c} \lambda C(j,i)/\sum_{j}\text{C}(j,i)\;\text{if}\;\sum_{j}\text{C}(j,i)\neq 0\\ \;0\;\text{otherwise} \end{array}$$ (6)

Let *u*_0_ and *v*_0_ be the initial probability vectors of the integrated multigraph MFSN and DPN, respectively. The initial probability vector of the CHN can then be represented as ${\text{P}}_{0}=\left[\begin{array}{c} (1-\eta )u_{0}\\ \eta v_{0} \end{array}\right]$. The parameter *η* ∈ (0,1) weighs the importance of the integrated multigraph MFSN and DPN. The initial probability of the integrated multigraph MFSN *u*_0_ is constructed such that equal probabilities are assigned to all of the seed nodes with the sum of the probabilities equal to 1. Similarly, the initial probability of the DPN *v*_0_ can be obtained.

Finally, we substituted the transition matrix *M* and initial probability P_0_ into the iterative equation (Eq 1). After a few steps, a stable probability vector ${\text{P}}_{\infty }=\left[\begin{array}{c} (1-\eta )u_{\infty }\\ \eta v_{\infty } \end{array}\right]$ can be obtained. All candidate miRNAs can now be ranked according to *u*_∞_, and the top ranked miRNAs can be considered as having a high probability of being associated with the disease of interest.

## Results

### Performance of CHNmiRD

For simplicity, we chose the following parameters to assess the performance of CHNmiRD in identifying potential miRNA-disease associations: *α* = 0.7 and *λ* = *η* = 0.5. The effect of these parameters was examined in the next section. 5-fold cross validation analysis of 3,462 known experimentally verified miRNA-disease associations, including 69 diseases associated with no less than 5 miRNAs, was used for this assessment. For a given disease *d*, the known experimentally verified miRNAs associated with disease *d* were randomly divided into 5 subsets. One subset was used as testing case, while the known disease *d*-related miRNAs in the rest sets and disease *d* were used as seed nodes in the multigraph MFSN and DPN, respectively. The candidate miRNAs included all of the miRNAs without known associations with disease *d*. We tested how well this testing case ranked relative to the candidate miRNA set for the given disease *d*. If the ranking of the testing miRNA exceeded a given threshold, this experimentally verified miRNA-disease association was considered to be successfully predicted by CHNmiRD.

**Fig 2:**
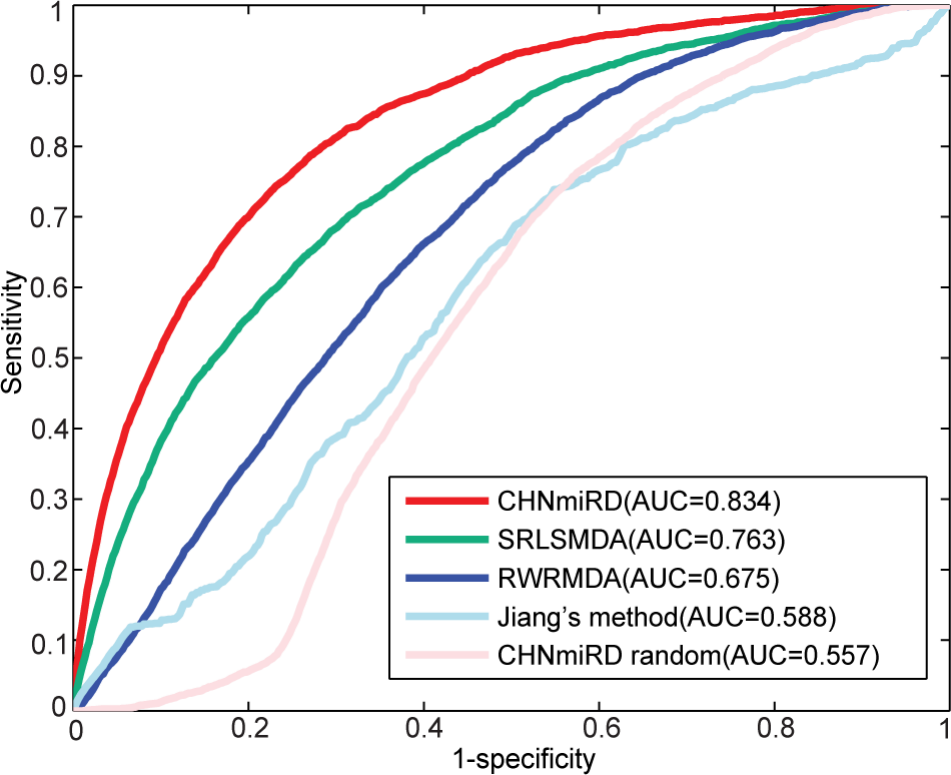
ROC curves and AUC values of CHNmiRD and other similar methods for 5-fold cross validation.

The ROC curve is a plot of the true positive rate (sensitivity) against the false positive rate (1-specificity) for different thresholds. Suppose TP denotes true positive, TN denotes true negative, FN denotes false negative, and FP denotes false positive, then the sensitivity is calculated as TP/(TP+FN), and specificity is calculated as TN/(TN+FP). Sensitivity refers to the proportion of the testing miRNAs ranked higher than a given threshold, and specificity refers to the proportion of the testing miRNAs ranked lower than this given threshold. We plotted an ROC curve by varying the threshold and calculated the value of the area under the ROC curve (AUC). AUC values range from 0 to 1, with 0.5 and 1.0 indicating random and perfect predictive performance, respectively. CHNmiRD achieved an AUC value of 0.834 when testing 3,462 known experimentally verified miRNA-disease associations (Fig 2). To examine whether the result generated by chance, the seed miRNAs were randomly selected from candidate miRNAs for each disease and the AUC value was calculated (Fig 2). The results indicated that the real AUC value was much higher than that in randomization tests. 19 human diseases which are associated with at least 50 miRNAs were also evaluated. As shown in Table 1, lung cancer achieved the highest AUC value while systemic lupus erythematosus had the lowest one. The average AUC value of these 19 diseases was 0.844. These results demonstrated that CHNmiRD was effective in recovering known experimentally verified miRNA-disease associations.

**Table 1:** AUC values of CHNmiRD and other similar methods for 19 human diseases using 5-fold cross validation.

Disease name	MIM ID	No. miR	CHNmiRD	Jiang’s	RWRMDA	SRLSMDA
Lung cancer	211980	208	0.920	0.589	0.777	0.832
Breast cancer	114480	229	0.911	0.573	0.777	0.913
Colorectal cancer	114500	239	0.904	0.557	0.807	0.909
osteosarcoma	259500	54	0.900	0.664	0.685	0.810
Hepatocellular cancer	114550	243	0.895	-	0.779	0.819
Pancreatic cancer	260350	127	0.876	0.648	0.691	0.844
Bladder cancer	109800	106	0.875	0.567	0.701	0.817
Esophageal cancer	133239	171	0.873	0.568	0.737	0.868
Glioblastoma	137800	155	0.872	0.563	0.744	0.857
Melanoma	155600	175	0.858	0.590	0.709	0.840
Prostate cancer	176807	148	0.857	0.576	0.725	0.854
nasopharyngeal cancer	607107	51	0.848	0.711	0.627	0.697
kidney cancer	144700	125	0.833	0.579	0.735	0.820
Thyroid cancer	188550	58	0.828	0.622	0.628	0.785
Acute myeloid leukemia	601626	86	0.822	-	0.575	0.611
Cervical cancer	603956	64	0.820	0.583	0.630	0.785
Medulloblastoma	155255	76	0.786	0.556	0.669	0.780
Adrenal cortical carcinoma	202300	67	0.777	0.625	0.617	0.677
Systemic lupus erythematosus	152700	83	0.711	-	0.594	0.622

Note: ‘No.miR’ indicates the number of miRNAs associated with a disease. ‘-’ denotes the disease- miRNA associations could not be predicted by Jiang’s method because of the lack of data.

To further evaluate the performance of individual data sources, we performed the same prediction framework by substituting the MFSN based on individual data sources for the integrated MFSN. The results are shown in Table 2. Although the PPI obtained the highest AUC value (0.817) among the four data sources, it was lower than that of the integrated method (0.834). The results showed that prediction performance improved upon integration of different genomic data sources. In addition, the coverage of miRNAs was different and biased for individual data sources. Therefore, some known disease-related miRNAs were ignored in the prediction process when using individual data sources. For example, 8 of 370 disease-related miRNAs were absent in the MFSN constructed based on BP ontology, and 36 of 370 disease-related miRNAs could not be prioritized when using CC ontology. Compared with individual data sets, the combined algorithm produced a higher coverage of miRNAs, which could be preferable for searching for novel disease-related miRNAs.

**Table 2:** Performance of individual data source.

Data source	PPI	BP	MF	CC
**AUC**	0.817	0.771	0.765	0.751
**No. of missing disease-related miRNAs**	0	8	17	36

### Robustness of CHNmiRD

To evaluate the robustness of CHNmiRD, we considered different miRNA targets, miRNA-disease associations, DPNs and parameters. The predicted targets of 402 miRNAs were obtained from TargetScan (version 6.2) [37], miRDB (version 5.0) [38] and TargetMiner (May 2012) [39] (see S5 File). CHNmiRD was implemented for 5-fold cross validation. As a result, an AUC value of 0.832 was achieved (S1 Fig), which was comparable with that of the experimentally verified targets. To examine whether CHNmiRD was sensitive to the miRNA-disease associations, we randomly removed the miRNA-disease associations from 5% to 30% with a step of 5%. The results showed that the number of miRNA-disease associations had slight effect on the results (S2 Table). Additionally, we constructed DPNs using 3-NN network and 7-NN network, and CHNmiRD was then performed. As a result, the AUC values of 0.833 and 0.834 were obtained for 3-NN network and 7-NN network using 5-fold cross validation (S2 Fig). This was comparable with that of 5-NN network (0.834), demonstrating that CHNmiRD was robust to the selection of K for the KNN network. CHNmiRD included three parameters: (1) the restart probability *α*; (2) the jumping probability *λ*; and (3) the parameter *η* which controlled the effect of the two seed nodes, seed miRNAs and seed diseases. Based on previous studies demonstrating that the predictive result was robust to the restart probability, parameter *α* was selected to be 0.7 [40–42]. To investigate the possible effects of parameters *λ* and *η* on the performance of CHNmiRD, various values were used for these two parameters, and 5-fold cross validation was performed. The AUC values for different combinations of these two parameters are shown in Table 3. The results of the validation showed that parameter *η* had only a slight effect on the performance, while an increase of parameter *λ* improved performance. Specifically, when parameter *λ* was in the range of 0.5 to 0.9, performance became stable and performed better. Thus, the dependence of this method on these two parameters is minimal, particularly when the value of *λ* is above 0.5.

**Table 3:** AUC values for different combinations of the two parameters.

	*η*				
*λ*	0.1	0.3	0.5	0.7	0.9
0.1	0.706	0.714	0.723	0.732	0.743
0.3	0.800	0.800	0.800	0.799	0.801
0.5	0.835	0.834	0.834	0.832	0.831
0.7	0.850	0.849	0.849	0.848	0.846
0.9	0.856	0.856	0.857	0.856	0.855

### CHNmiRD versus similar existing methods

To further demonstrate the advantages of CHNmiRD in identifying miRNA-disease associations, we compared our model with the following similar existing methods: Jiang’s method [15], RWRMDA [16] and SRLSMDA [19]. Jiang et al. [15] adopted a hypergeometric distribution model for inferring potential miRNA-disease associations based on the human phenome-miRNAnome network. Chen et al. proposed two methods to uncover the relationships between miRNAs and diseases: RWRMDA and SRLSMDA. RWRMDA [16] used random walk method on the MFSN, while SRLSMDA [19] combined the optimal classifiers in disease space and miRNA space using regularized least squares method. We applied the RWRMDA to the integrated multigraph MFSN and applied Jiang’s method and SRLSMDA to the CHN, respectively. 5-fold cross validation was then performed using the same dataset. The best parameters were selected for other prediction methods (see S6 File) and the AUC values were obtained (see Fig 2 and Table 1). As the results indicated, CHNmiRD (AUC = 0.834) performed better than Jiang’s method (AUC = 0.588), RWRMDA (AUC = 0.675) and SRLSMDA (AUC = 0.763).

Additionally, there were some diseases without known related miRNAs and the pathological mechanism of these diseases at the miRNA level was completely unknown. A recent study indicated that SRLSMDA showed a better performance for this kind of disease [19]. We therefore tested the efficacy of CHNmiRD in searching miRNA-disease associations for these diseases. In the DPMN, 105 diseases were connected to 370 miRNAs. For each of these 105 diseases, we removed all of the relationships of this disease to miRNAs and used this disease as a seed node to implement CHNmiRD and RLSMDA. If one of the known disease-related miRNAs was ranked in the top N of the ranked list, we considered it to be a successful prediction. Here, we set N as 1, 5, 10, 20 and 50. As indicated in Table 4, CHNmiRD successfully ranked 40 miRNAs as top 1, while SRLSMDA only ranked 14 miRNAs as top 1. Moreover, CHNmiRD performed better than SRLSMDA as N varied.

**Table 4:** The number of successfully predicted miRNAs with different Ns.

Top N	Top 1	Top 5	Top 10	Top 20	Top 50
**SRLSMDA**	14	80	140	280	779
**CHNmiRD**	40	138	249	434	987

### Case studies

To illustrate the application of CHNmiRD in identifying novel disease-related miRNAs, case studies of glioblastoma (GBM), myocardial infarction (MI) and type 1 diabetes (T1D) considering different available numbers of seed miRNAs were examined. For a given disease, the known miRNAs associated with that disease were referred to as seed miRNAs. Based on the aforementioned known miRNA-disease associations, GBM had 155 seed miRNAs, MI had 40 seed miRNAs, and T1D had 1 seed.

For each of these three diseases, all of the candidate miRNAs (non-seed miRNAs) were ranked based on CHNmiRD (S3 Table), and the top 10 predicted miRNAs in the ranked list were examined. Because the known miRNA-disease associations were collected from the HMDD database, which was last updated in 2013, we manually verified these miRNA-disease associations by checking more recently published literatures. The results are illustrated in Table 5. Ten, 8 and 3 of the top 10 predicted miRNAs were confirmed in GBM, MI and T1D, respectively, according to recently reported biological experiments, and almost all of these had high ranks in the predicted miRNA lists. Although the remaining 9 predicted miRNA-disease associations had not yet been validated directly, these associations could be interpreted indirectly by recent studies. For instance, gene expression profile analysis of patient whole blood revealed that hsa-miR-182-5p was deregulated in patients with coronary artery disease [43]. Additionally, hsa-miR-19b-3p was reported to be a potential anti-thrombotic protector in patients with unstable angina [44], which has a high probability of developing into acute myocardial infarction. The remaining 7 predicted miRNAs which were not validated to be associated with T1D directly were found to be associated with diabetes [45, 46] and type 2 diabetes (T2D) [47–49]. It is worth noting that T1D had only one seed miRNA, but CHNmiRD achieved excellent performance. Collectively, these results not only indicated the reliability of CHNmiRD in identifying novel disease-associated miRNAs, but also demonstrated its potential application value in biomedical research.

**Table 5:** Literature evidence for top 10 miRNAs of glioblastoma, myocardial infarction and type 1 diabetes.

miRNA	Rank	Literature validation	PubMed ID	Year
**Glioblastoma**				
hsa-miR-200a-3p	1	Yes/directly	24755707	2014
hsa-miR-190a-5p	2	Yes/directly	23863200	2013
hsa-miR-126-3p	3	Yes/directly	21713760	2012
hsa-miR-126-5p	4	Yes/directly	21713760	2012
hsa-miR-223-3p	5	Yes/directly	24438238	2014
hsa-miR-29b-3p	6	Yes/directly	24155920	2013
hsa-miR-34c-5p	7	Yes/directly	24140020	2013
hsa-miR-34b-5p	8	Yes/directly	24213470	2012
hsa-miR-1-3p	9	Yes/directly	24310399	2014
hsa-miR-34b-3p	10	Yes/directly	24213470	2012
**Myocardial infarction**				
hsa-miR-146a-5p	1	Yes/directly	23208587	2013
hsa-miR-17-5p	2	Yes/directly	24900964	2014
hsa-miR-17-3p	3	Yes/directly	24900964	2014
hsa-miR-125b-2-3p	4	Yes/directly	24627568	2014
hsa-miR-125b-5p	5	Yes/directly	24627568	2014
hsa-miR-182-3p	6	No/ indirectly	-	-
hsa-miR-19b-3p	7	No/ indirectly	-	-
hsa-miR-34c-5p	8	Yes/directly	23047694	2012
hsa-miR-29c-3p	9	Yes/directly	20164119	2010
hsa-miR-29c-5p	10	Yes/directly	24900964	2014
**Type 1 diabetes**				
hsa-miR-155-5p	1	Yes/directly	24223694	2013
hsa-miR-16-5p	2	No/ indirectly	23233752	2013
hsa-miR-146a-5p	3	Yes/directly	24796653	2014
hsa-miR-15a-5p	4	No/ indirectly	24397367	2014
hsa-miR-21-5p	5	Yes/directly	24937532	2014
hsa-miR-15a-3p	6	No/ indirectly	24397367	2014
hsa-miR-17-5p	7	No/ indirectly	22960330	2012
hsa-miR-16-1-3p	8	No/ indirectly	23233752	2013
hsa-miR-96-5p	9	No/ indirectly	24981880	2014
hsa-miR-128-3p	10	No/ indirectly	24944010	2014

## Discussion

In this work, a computational framework, CHNmiRD, was presented for the prediction of novel miRNA-disease associations by integrating multiple genomic and phenotype data. Based on PPI data and GO data (three sub-ontologies: BP, MF and CC), four MFSNs were constructed using miRNA-target relationships and were further merged in to an integrated multigraph MFSN. A CHN was then constructed by connecting the integrated multigraph MFSN and DPN using the known miRNA-disease relationship information. Finally, novel miRNA-disease associations were predicted by implementing a global network distance measure-based random walk analysis on the CHN.

Comparing the integrated data with the individual data sources using the same method, we found that PPI data was the most effective in prioritizing candidate miRNAs among the four data sets. However, the performance of PPI data was inferior to the combined method, because individual data tend to be incomplete and noisy. In addition, the combined method covered more miRNAs, which was favorable for uncovering novel disease-related miRNAs.

The results of cross validation indicated the improved performance of CHNmiRD over other similar existing methods, especially for diseases without any known associated miRNAs. In addition, CHNmiRD did not need negative samples and the performance became stable and performed better when parameter *λ* was in the range of 0.5 to 0.9. Furthermore, case studies demonstrated the reliability and effectiveness of this method in revealing novel disease-related miRNAs. Each of the top 10 miRNAs in the three case studies was either directly or indirectly validated by recently published research. It worth noting that we did not compare CHNmiRD with our previously described method [21] because of different data sources used in the two methods. Moreover, the known disease-miRNA associations were not used in our previous method, thus the cross validation could not be implemented.

The CHNmiRD is based on the CHN, and thus the efficacy of CHNmiRD is affected by the quality of the CHN. For future studies, more bioinformatics data should be integrated to improve the quality of the CHN. For example, expression profile and/or pathway data can be added into the integrated MFSN, and the similarity of disease phenotypes based on ontological descriptions can also be added into the DPN. We anticipate that our algorithm may be more comprehensive and effective with the increasing amount of available miRNA-related biological data. In summary, CHNmiRD could potentially provide an improved tool for predicting novel miRNA-disease associations and play an important role in deciphering the pathogenesis of complex human diseases at the post-transcriptional level.

## Supporting Information

S1 Fig The ROC curve and AUC value of CHNmiRD with predicted miRNA targets for 5-fold cross validation. (TIF)

S2 Fig The ROC curve and AUC value of CHNmiRD with different DPNs for 5-fold cross validation. (A). The DPN constructed based on 3-NN network. (B). The DPN constructed based on 7-NN network.
(TIF)

S1 File Known human miRNA-disease associations. (XLS)

S2 File Experimentally validated miRNA targets. (XLS)

S3 File The DPN constructed using 5-NN network. (XLS)

S4 File The integrated multigraph MFSN. (XLSX)

S5 File Obtaining the predicted miRNAs targets. (DOC)

S6 File Parameters selection of other miRNA-disease association prediction methods. AUC values of RWRMDA for 5-fold cross validation with variation of the parameter (Table A). AUC values of SRLSMDA for 5-fold cross validation with variation of the parameter (Table B).
(DOC)

S1 Table Comparison of different methods for inferring miRNA-disease associations. (DOC)

S2 Table AUC values of CHNmiRD for 5-fold cross validation with variation of the number of miRNA-disease associations. (DOC)

S3 Table The candidate miRNA lists for glioblastoma, myocardial infarction and type 1 diabetes based on CHNmiRD. (XLS)
