# Supplementary figures and images for: Integration of Multiple Genomic and Phenotype Data to Infer Novel miRNA-Disease Associations
Source: PLoS One. 2016 Feb 5;11(2):e0148521. doi: 10.1371/journal.pone.0148521 (PMC4743935; doi:10.1371/journal.pone.0148521)

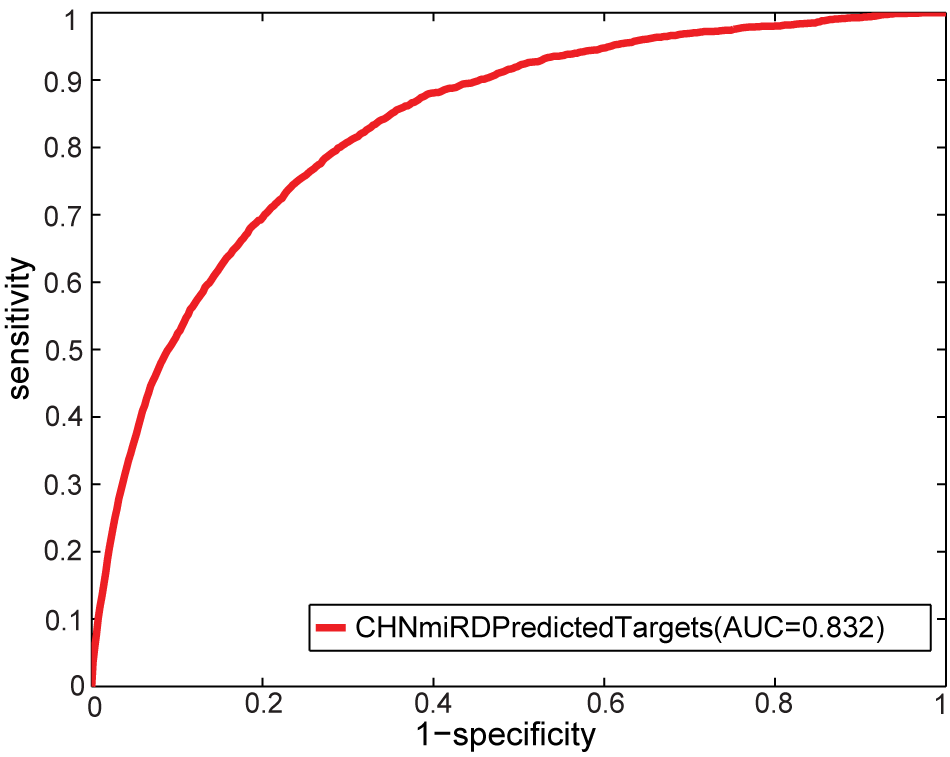

Supplement: S1 Fig — (TIF) [file pone.0148521.s001.tif]

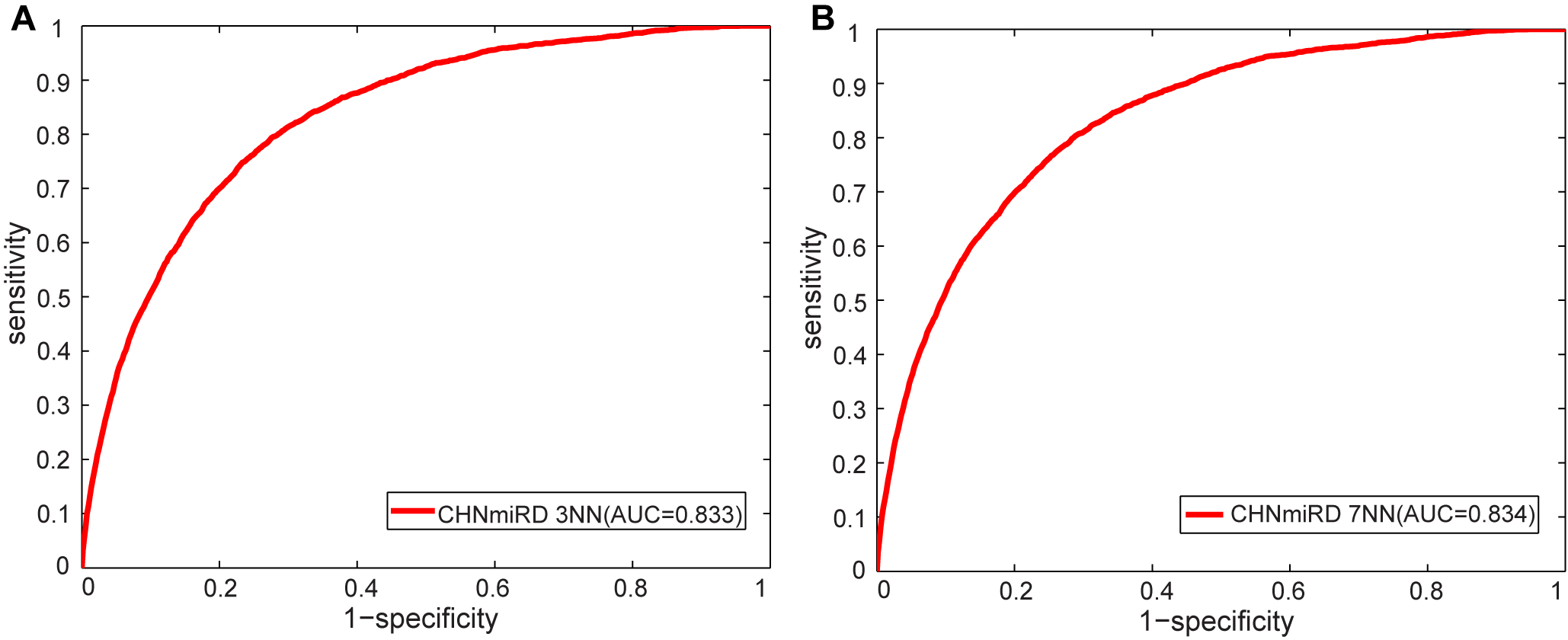

Supplement: S2 Fig — (A). The DPN constructed based on 3-NN network. (B). The DPN constructed based on 7-NN network. (TIF) [file pone.0148521.s002.tif]
