# Supplementary material for: Integration of Multiple Genomic and Phenotype Data to Infer Novel miRNA-Disease Associations
Source: PLoS One. 2016 Feb 5;11(2):e0148521. doi: 10.1371/journal.pone.0148521 (PMC4743935; doi:10.1371/journal.pone.0148521)
Supplement: S5 File — (DOC) [file pone.0148521.s007.doc]

**Obtaining the predicted miRNAs targets**

The predicted targets of 402 miRNAs were obtained from TargetScan (version 6.2, Jun 2012) [1], miRDB (version 5.0, Aug 2014) [2] and TargetMiner (May 2012) [3]. To increase the reliability of the results, the regulatory associations between miRNAs and targets appeared in at least two databases were retained in our study. In total, we obtained 125,582 targeting pairs that involved 397 miRNAs and 10,260 target genes.

**Supplementary references**

1. Grimson A, Farh KK, Johnston WK, Garrett-Engele P, Lim LP, Bartel DP. MicroRNA targeting specificity in mammals: determinants beyond seed pairing. Mol Cell. 2007;27(1):91-105. PubMed PMID: 17612493.

2. Wang X. miRDB: a microRNA target prediction and functional annotation database with a wiki interface. Rna. 2008;14(6):1012-7. PubMed PMID: 18426918.

3. Bandyopadhyay S, Mitra R. TargetMiner: microRNA target prediction with systematic identification of tissue-specific negative examples. Bioinformatics. 2009;25(20):2625-31. PubMed PMID: 19692556.
