# Supplementary material for: Integration of Multiple Genomic and Phenotype Data to Infer Novel miRNA-Disease Associations
Source: PLoS One. 2016 Feb 5;11(2):e0148521. doi: 10.1371/journal.pone.0148521 (PMC4743935; doi:10.1371/journal.pone.0148521)
Supplement: S6 File — AUC values of RWRMDA for 5-fold cross validation with variation of the parameter (Table A). AUC values of SRLSMDA for 5-fold cross validation with variation of the parameter (Table B). (DOC) [file pone.0148521.s008.doc]

**Parameters selection of other miRNA-disease association prediction methods**

In this study, we compared our model (CHNmiRD) with Jiang’s method [1], RWRMDA [2] and SRLSMDA [3]. There is no parameter in Jiang’s method. There is one parameter (restart probability$\gamma$) in RWRMDA [2] and 3 parameters (two trade-off parameters$\eta_{M}$,$\eta_{D}$ and one weight parameter$\omega$) in SRLSMDA [3]. According to previous literatures[3, 4], we chose $\eta_{M}=1$ and $\eta_{D}=1$. In order to select the best parameters for each of these two methods, 5-fold cross validation was implemented, and the AUC values for different parameters were obtained. As shown in Tables A and B in S6 File, RWRMDA and SRLSMDA achieved the best performance when the parameter of $\gamma$ was set as 0.9 and $\omega$ was set as 0.3. Therefore, we selected$\gamma=0.9$and$\omega=0.3$.

## Table A. AUC values of RWRMDA for 5-fold cross validation with variation of the parameter.

| **Restart probability** $\gamma$ | **0.1** | **0.3** | **0.5** | **0.7** | **0.9** |
| --- | --- | --- | --- | --- | --- |
| **AUC** | 0.644 | 0.651 | 0.665 | 0.666 | 0.675 |

## Table B. AUC values of SRLSMDA for 5-fold cross validation with variation of the parameter.

| **Weight parameter** $\omega$ | **0.1** | **0.3** | **0.5** | **0.7** | **0.9** |
| --- | --- | --- | --- | --- | --- |
| **AUC** | 0.762 | 0.763 | 0.762 | 0.760 | 0.753 |

**Supplementary references**

1. Jiang Q, Hao Y, Wang G, Juan L, Zhang T, Teng M, et al. Prioritization of disease microRNAs through a human phenome-microRNAome network. BMC Syst Biol. 2010;4 Suppl 1:S2. PubMed PMID: 20522252.

2. Chen X, Liu MX, Yan GY. RWRMDA: predicting novel human microRNA-disease associations. Mol Biosyst. 2012;8(10):2792-8. PubMed PMID: 22875290.

3. Chen X, Yan GY. Semi-supervised learning for potential human microRNA-disease associations inference. Sci Rep. 2014;4:5501. PubMed PMID: 24975600.

4. van Laarhoven T, Nabuurs SB, Marchiori E. Gaussian interaction profile kernels for predicting drug-target interaction. Bioinformatics. 2011;27(21):3036-43. PubMed PMID: 21893517.
