# Supplementary material for: Integration of Multiple Genomic and Phenotype Data to Infer Novel miRNA-Disease Associations
Source: PLoS One. 2016 Feb 5;11(2):e0148521. doi: 10.1371/journal.pone.0148521 (PMC4743935; doi:10.1371/journal.pone.0148521)
Supplement: S1 Table — (DOC) [file pone.0148521.s009.doc]

## S1 Table. Comparison of different methods for inferring miRNA-disease associations.

| **Method** | **Inputs** | **Outputs** | **Limitations** |
| --- | --- | --- | --- |
| **Jiang’s method** | (1) (2) (3) | Predicted 514 miRNAs list for each of the 1599 diseases. | (5) (6) |
| **RWRMDA** | (2) (4) | Predicted 271 miRNAs list for any disease with known related miRNAs. | (7) (8) |
| **NetCBI** | (1) (2) (3) | Predicted 271 miRNAs list for each of the 5080 diseases. | (8) |
| **HDMP** | (2) (4) | Predicted 338 miRNAs list for each of the 5080 diseases. | (7) (8) |
| **RLSMDA** | (1) (2) (3) | Predicted 271 miRNAs list for each of the 137 diseases. | (8) |

Note: (1) disease phenotype network (DPN), (2) MFSN, (3) known miRNA-disease associations, (4) The known miRNAs associated with the disease of interest, (5) The MFSN they constructed considered the number of overlapping miRNA targets while neglecting the functional link between them. (6) The method was not work for disease whose all neighbor diseases (in the DPN) are not associated with any known miRNAs. (7) The method was not applicable to disease which did not have any known related miRNAs. (8) The same miRNA-disease relations were used to construct the MFSN and evaluate the performance, which might over-estimate the performance.
