# Supplementary material for: Integration of Multiple Genomic and Phenotype Data to Infer Novel miRNA-Disease Associations
Source: PLoS One. 2016 Feb 5;11(2):e0148521. doi: 10.1371/journal.pone.0148521 (PMC4743935; doi:10.1371/journal.pone.0148521)
Supplement: S2 Table — (DOC) [file pone.0148521.s010.doc]

## S2 Table. AUC values of CHNmiRD for 5-fold cross validation with variation of the number of miRNA-disease associations.

| **Percentage of removed** | **5%** | **10%** | **15%** | **20%** | **25%** | **30%** |
| --- | --- | --- | --- | --- | --- | --- |
| **AUC** | 0.820 | 0.808 | 0.802 | 0.791 | 0.792 | 0.785 |
